# Cross-cultural comparison of beauty judgments in visual art using machine learning analysis of art attribute predictors among Japanese and German speakers

**DOI:** 10.1038/s41598-024-65088-z

**Published:** 2024-07-10

**Authors:** Jan Mikuni, Blanca T. M. Spee, Gaia Forlani, Helmut Leder, Frank Scharnowski, Koyo Nakamura, Katsumi Watanabe, Hideaki Kawabata, Matthew Pelowski, David Steyrl

**Affiliations:** 1 Vienna Cognitive Science Hub, University of Vienna, Kolingasse 14-16, 1090 Vienna, Austria; 2 Department of Neurology, Center of Expertise for Parkinson and Movement Disorders, Donders Institute for Brain, Cognition and Behavior, Radboud University Medical Centre, Nijmegen, The Netherlands; 3 Department of Cognition, Emotion, and Methods in Psychology, Faculty of Psychology, University of Vienna, Vienna, Austria; 4 Department of Rehabilitation, Donders Institute for Brain, Cognition and Behavior, Center of Expertise for Parkinson and Movement Disorders, Radboud University Medical Centre, Nijmegen, The Netherlands; 5 Faculty of Science and Engineering, Waseda University, Tokyo, Japan; 6 Department of Psychology, Faculty of Letters, Keio University, Tokyo, Japan

**Keywords:** Psychology, Human behaviour

## Abstract

In empirical art research, understanding how viewers judge visual artworks as beautiful is often explored through the study of attributes—specific inherent characteristics or artwork features such as color, complexity, and emotional expressiveness. These attributes form the basis for subjective evaluations, including the judgment of beauty. Building on this conceptual framework, our study examines the beauty judgments of 54 Western artworks made by native Japanese and German speakers, utilizing an extreme randomized trees model—a data-driven machine learning approach—to investigate cross-cultural differences in evaluation behavior. Our analysis of 17 attributes revealed that visual harmony, color variety, valence, and complexity significantly influenced beauty judgments across both cultural cohorts. Notably, preferences for complexity diverged significantly: while the native Japanese speakers found simpler artworks as more beautiful, the native German speakers evaluated more complex artworks as more beautiful. Further cultural distinctions were observed: for the native German speakers, emotional expressiveness was a significant factor, whereas for the native Japanese speakers, attributes such as brushwork, color world, and saturation were more impactful. Our findings illuminate the nuanced role that cultural context plays in shaping aesthetic judgments and demonstrate the utility of machine learning in unravelling these complex dynamics. This research not only advances our understanding of how beauty is judged in visual art—considering self-evaluated attributes—across different cultures but also underscores the potential of machine learning to enhance our comprehension of the aesthetic evaluation of visual artworks.

## Introduction

“Beauty is not a quiet thing, rather it can pierce you, it can paralyze you and it can lead you to the dimension of the sublime.”

Umberto Galimberti (quote from lecture: “Beauty as secret rule of life”; VII Festival Vacanze dell'Anima-Movimenti di bellezza, Museo Canova di Possagno, 21/07/2016. Free translation).

When encountering works of visual art, individuals assess these along various aesthetic judgments such as personal preference, creativity, or subjectively evaluated quality. Among these aesthetic judgments, beauty has been recognized as one of the most prototypical^1^, historically engaging multiple disciplines, including philosophy, anthropology, history, the arts, and psychology, for centuries^2–6^. Despite its longstanding discourse, beauty remains a complex, multifaceted concept that eludes a universal definition and continues to foster ongoing debate.

The present study recognises aesthetic judgments as value assignments individuals attribute to aesthetic products, such as visual artworks. These judgments are suggested to be shaped by an intricate mix of perceptual, cognitive, and socio-cultural influences and might show interindividual similarities or differences^7–9^. Specifically, aesthetic judgments such as beauty, liking, and creativity are not merely responses to the sensory properties of artworks; they are deeply rooted in the viewers’ perceptual and cognitive processes and their socio-cultural backgrounds. These processes, as outlined by Berlyne^6,10–13^, Chatterjee^14,15^, and Leder and colleagues establishing a model of aesthetic appreciation and judgments^7,8,16^, involve multiple stages. These stages start from an initial sensory perception of artworks’ qualities to integrating this information during intermediate stages into emotional and cognitive processing and culminating in a sophisticated evaluation process, where meanings are ascribed and judgments—such as beauty—are formed. Hence, these judgments are not isolated assessments but are influenced by—and argued along with—attributes such as color usage, visual harmony, emotional valence (negative or positive), level of symbolism and abstractness, or emotional expressiveness, among others^14^.

Our current study is built on prior work and analysis^17,18^, assessing the relationship between aesthetic judgements and art attributes to underscore how individuals formulate certain aesthetic judgements based on data-driven approaches. Spee and colleagues^17^ investigated the judgment of creativity using a machine-learning approach. They found that art attributes such as symbolism, emotional expressiveness, and imaginativeness of the contents significantly predict the evaluated creativity judgment of artworks in a German-speaking population. The following publication^18^ used again a machine-learning approach to explore the relationship between various aesthetic judgments—including beauty judgments—and attributes, establishing a methodological data-driven framework. The results showed that art attributes significantly predicted beauty judgments, specifically valence, visual harmony, emotional expressiveness, simplicity, depth (2D vs. 3D), and color variations.

Following this pioneering work, the present study further investigated, as a next step, how socio-cultural backgrounds might influence this result, focusing on beauty judgments. To extend our research, we used the existing dataset from the German-speaking population of Spee et al.^17,18^ and collected additional data in Japan with a Japanese-speaking sample to enable the cross-cultural comparison of beauty judgments. The cross-cultural component of our study is particularly significant as it addresses how cultural backgrounds influence the perception and evaluation of beauty in visual art. Studies in art and aesthetic research have emphasized the impact of viewers’ cultural backgrounds on their interactions with and evaluations of artworks^19–21^. Cultural influences might arise from differences in education, language, social systems, and a lifetime of exposure to diverse visual features and forms, which can ultimately influence perceptual and cognitive processes^30^. Such debate illuminates beauty as a prevalent judgment in art, but how such socio-cultural background could influence the association between beauty judgments and the specific attributes remains underexplored. This gap is especially pronounced in cross-cultural contexts, where diverse cultural appreciations and evaluations of visual art suggest a complex interplay between intercultural commonalities and culturally specific assessments of beauty.

Our current study examined how subjectively evaluated art attributes influence beauty judgments, focusing on two cultural cohorts, operationalized as European (Austria, German-speaking) and East Asian samples (Japan, Japanese-speaking). The two cultural cohorts were selected as the comparisons between European and East Asian samples have been extensively assessed in past research. Hence, we can understand our findings in parallel with past studies. We used the same set of 17 art attributes as used in the prior studies^17,18^, which based the selection mainly on the assessment of art attributes^14^ (AAA). This list of attributes acknowledges the processing stages of the model of aesthetic judgments (see for a review, methodological justifications, and limitations, Spee and colleagues^18^).

Our study seeks to answer the following research questions: (1) Which attributes in visual artworks and, correspondingly, which cognitive stages contribute to the judgment of beauty? Note that the latter is argued as a theoretical discussion along the model of art appreciation and art judgments^7^ to provide a list of implications for future research. (2) Does cultural background, operationalized as the difference between Japanese and German native speakers, significantly affect beauty judgments? Moreover, if so, what are the intercultural differences and commonalities in the art attributes that predict beauty? Importantly, our study does not aim to define beauty per se; instead, we aim to understand the associations between beauty judgments and art attributes and the impact of cultural background on this relationship.

As introduced in the previous work^17,18^, we used a data-driven approach with machine learning, i.e., extreme randomized trees (ET)^22^, and employed a three-step analysis to answer the questions. In Step 1, we assess whether machine learning can predict beauty ratings from art attributes and evaluate the influence of viewers’ cultural backgrounds. In Step 2, we determine which art attributes significantly contribute to beauty judgments within Japanese- or German-speaking populations separately. Finally, in Step 3, we compare the results from Step 2 to uncover differences in the art attributes influencing beauty ratings between these two groups. With this exploratory approach, encompassing multiple attributes and broadening the scope from linear, hypothesis-driven to non-linear data-driven statistical approaches^23,24^, we intend to provide new insights into the intricate nature of aesthetic judgments, particularly in the context of beauty in visual art.

## Material and methods

### Sample size

Determining the exact number of samples required for data analysis using complex machine-learning techniques remains a debated topic. To the best of our knowledge, there are no established standards. Considering this, we adhered to various recommended practices for selecting an appropriate sample size. Firstly, at least 50 samples are needed to initiate substantial machine learning analysis^25^. Secondly, having 10 to 20 samples for each degree of freedom (such as predictors or attributes in the analysis) is considered adequate, especially logistic regression^26^. Thirdly, a conventional power analysis employing a two-tailed Student’s *t*-test, with an alpha of 0.05 and a power of 0.8, indicates that 394 samples are necessary to identify more minor effect sizes^27–29^. Lastly, as with other statistical approaches, a larger sample size enhances the capability of discerning more minor effects. We note that samples here do not refer to the number of participants but rather the number of the acquired responses. We have 102 native Japanese speakers and 83 native German speakers, rating all 54 paintings. Hence, for Step 1 in our analysis, we have 10,071 samples of beauty ratings in total. For Step 2, we have 5486 and 4585 samples for native Japanese and German cohorts, respectively. Hence, our sample size should be able to detect minor effects. Although our sample size is larger than what is required from the power analysis, we chose to have a larger sample size to ensure the robustness of our findings, given the complexity of the factors being studied in the present study.

### Participants

This study involved a final sample of 102 native Japanese students (56 female, *M*_*age*_ = 21.28, *SD* = 2.10, Japanese group in the following text) from Waseda University (Tokyo, Japan) and 83 native German-speaking students from the University of Vienna in Austria (58 female, *M*_*age*_ = 23.79, *SD* = 3.43, German group in the following text). The data from 78 native German-speaking participants was retrieved from prior work^17,18^. Five more native German-speaking people were recruited to ensure higher statistical power. All participants had normal or corrected-to-normal vision. The final sample in both cultural groups was reduced from an initial collection of 143 and 92 participants from Waseda University and the University of Vienna, respectively, due to issues with data collection (i.e., some participants did not complete the online study, participant inputs were not recorded correctly); in addition, non-native speakers were excluded. In total, 41 participants from Waseda University and 9 participants from the University of Vienna were excluded from the following analysis.

The study setting had to be slightly different for the two groups: Due to the COVID-19 pandemic, it was not possible to conduct a study in person at Waseda University. Consequently, while the experiment was conducted in person in a testing room at the University of Vienna, the Japanese group from Waseda University joined an online study. Participants at Waseda University were recruited via the website of the university, while those at the University of Vienna were recruited via the participants’ pool of the university. Participants from Waseda University were paid 3,000 Japanese yen (approximately $30 US as of March 2020) for their participation, and those from the University of Vienna received course credits for their participation. The study was approved by the local ethical committee of Waseda University (Japan) as well as of the University of Vienna (Austria) following the declaration of Helsinki.

### Procedure

Participants from two cultural groups, i.e., Japanese and German, were included in this study. The Japanese group joined the study online via a URL link, where they gave informed consent before commencing. The German group, on the other hand, participated in person at the University of Vienna, providing written consent. Before starting the main experiment, all participants provided demographic information. They were instructed to rate a series of artworks using semantic differentials for various attributes. Each painting was presented individually, and participants could slide a 100-point Likert scale for each attribute. The order of artwork presentation and attribute scales was randomized. Participants were prompted to answer all questions. After the rating task, participants completed the Art Interest and Knowledge Questionnaire^30^. We note that the results of Art Interest and Knowledge Questionnaire are not the focus of the present study, and the questionnaires are not validated in the Japanese version used in the present study. For this reason, providing art interest and knowledge scores might not be able to draw meaningful information on participants’ demographic characteristics, and thereby, they are not reported in the results section.

### Stimuli and apparatus

The study employed 54 paintings selected from the Vienna Art Picture System (VAPS)^31^, ensuring a diverse representation of styles, content, and periods. The paintings included equal numbers of art motives (portrait, landscape, still-life) and art categories (representative, impressionist, abstract art). Ratings for each painting in the VAPS were checked for normal distribution, with most attributes found to be normally distributed except familiarity, see Spee et al.^17,18^ for detailed description and pre-ratings).

The main prediction target, the judgment of beauty, was assessed by asking participants, “How beautiful do you find the artwork?” The study also employed 17 art attributes as predictors, translated into Japanese by one of the first authors of the present study, who is a native Japanese speaker (see Art Attribute Selection, and for a detailed review on a selection of attributes, see prior research^17,18^). The German version was adapted from the previous research^17,18^.

The Japanese group used their laptops for the online study, resulting in variable screen sizes. The German group, participating in person at the University of Vienna, viewed paintings on a standardized 19" Iiyama ProLite B1906S display monitor, with the longest dimension of each artwork fixed at a maximum of 500 pixels, 1280 × 1024, 60 Hz resolution from a consistent distance of approximately 80 cm. The study was conducted using the online questionnaire tool Sosci Survey^32^.

### Art attributes selection

Regarding the art attributes used in the present study, we followed the previous studies by Spee and colleagues^17,18^. Specifically, they include *formal-perceptual* and *content-representational* dimension poles; following the Assessment of Art Attributes battery^14^, presented a most suitable and well-educated set of attributes. The AAA encompasses six *formal-perceptual* attributes (i.e., balance, color saturation, color temperature, depth, complexity, brushstroke) and six *conceptual-representational* attributes (i.e., abstractness, vividness, emotionality, imaginativeness, factual accuracy, symbolism). Following prior research^17,18^, we incorporated these attributes with minor modifications. In addition, the attributes were modified to align with the linguistic nuances of Japanese and German languages. Additionally, the scales were presented using the semantic differential technique, characterized by polar word pairs, as initially introduced by Adams and Osgood^33^. All attributes are shown in Table 1 (see for German and Japanese versions Supplementary Materials Tables S1, S2).

**Table 1:** 17 art attributes used in present study to predict beauty adopted from Spee et al.^17,18^.

Instructions	Please evaluate the artwork based on the different attributes	
Art attributes	Presented items with dimension poles to the participants	
	Negative pole (minimum value)	Positive pole (maximum value)
Formal perceptual attributes		
1. Visual harmony	Visual harmony, proportional	Peculiar, strange shapes
2. Depth	Two-dimensional	Three-dimensional
3. Complexity	Simple	Complex
4. Color saturation	Soft, pastel	Intense, strong
5. Color variety	Less colors	Many colors
6. Color temperature	Warm colors	Cold colors
7. Color world	Dark color world	Light color world
8. Brushwork	Fine brushwork	Rough brushwork
9. Utilization of drawing area	Little utilization of the Painting area	All utilization of the Painting area
Content representational attributes		
10. Abstraction	Representative	Abstract
11. Realism/Imaginativeness	Realistic content/topic	Imaginary, unreal, fantastic
12. Symbolism (ambiguity)	Distinct/clear (unambiguous, direct interpretation)	Symbolic (ambiguous, room for interpretation)
13. Accurate object representation	photorealistic	Painterly
14. Liveliness/animation	Dynamic	Still
15. Emotional expressiveness	Emotionless	Emotionally loaded
16. Valence	Negative valence	Positive valence
17. Focus	Much context/environment in the image	Focused content

### Interpretable machine learning based data analysis approach

To assess whether the art attributes significantly predict beauty and whether culture impacts beauty judgments, we conceived a three-steps analysis.

#### Step 1: Assessing the impact of 17 attributes and culture in predicting beauty

First, we examined whether the art attributes and the participants’ mother tongues are significant predictors of the beauty judgment. If our predictors do not explain a significant amount of the variance in the beauty judgments, any speculations on which attributes predict beauty and the difference between the two groups do not seem warranted.

To this aim, we trained a predictive regression model for the beauty scores using beauty scores from all participants as the prediction target and all 17 art attributes as well as the participant’s mother tongue (either Japanese or German) as predictors. Further, the contributions of predictors to the final scores of the beauty, e.g., how much the mother tongue of the art viewers contributed to predicting the beauty judgments, were examined with SHAP^34^ (Shapley Additive exPlanations^40^). The regression task itself was carried out with extreme randomized trees (ET) ensembles^22^. This machine learning method is computationally efficient and generally highly accurate since it can model non-linear relationships between predictors and prediction targets (beauty appraisals) as well as predictor interactions (Python v3.8.5, scikit-learn library v0.23.2)^22,35,36^.

The accuracy of regression predictions was evaluated through a nested cross-validation (CV) procedure^37^. This approach involves repeated train-test splits of the data to determine how well a model can adapt to new, unseen data. We employed shuffle-split repetitions with 128 iterations, allocating 20% of the data for testing and the remaining 80% for training within the primary CV loop. During each repetition, the training data was used to scale the data (using min–max scaling) and optimize the model’s complexity. The optimization of model complexity took place within a separate, nested CV process, which utilized a sequential Bayesian optimization technique along with a shuffle-split method (20% for testing, 80% for training, 128 iterations, starting with 64 initial configurations), so to identify the best-performing complexity parameters (BayesSearchCV, scikit-optimize, v0.8.1, min_samples_split 2 to n_samples, min_samples_leaf 1 to n_samples/2, max_features fraction 0 to 1). The best complexity parameters identified for maximum prediction accuracy in the training set were then applied, along with fixed parameters such as n_estimators = 333, criterion = friedman_mse and all other left to default, to train ET regressor models in the main CV loop. These models were later evaluated on the testing set designated for the main CV loop. The testing-set was not used in the inner CV loop. The performance of the regression models was assessed using the prediction coefficient of determination (prediction *R*^*2*^)^35^. *R*^*2*^ values are scaled such that a value of 0 indicates performance, which is equivalent to using the average target value as the predictor, also known as the trivial predictor. On the other hand, a value of 1 signifies perfect prediction accuracy without any errors. *R*^*2*^ values can indeed range from negative infinity, indicating a model’s performance is worse than merely using the target value as the prediction, while again 1 indicates a perfect model with no errors at all. We note that the value of prediction *R*^*2*^ will be smaller than the traditional *R*^*2*^ with conventional statistical models. This is due to the fact that the prediction *R*^*2*^ assessed the model’s ability to predict unseen data, thus focusing on the model’s predictive power rather than its fit to known data. The model analysis was carried out with SHAP^34^. SHAP is a method from interpretable machine learning and is based on Shapley values—a method from cooperative game theory—and measures the contributions of each predictor (here 17 art dimensions and the mother tongue of the participants) for a models’ prediction. Pooling those contributions over many single predictions allows a comprehensive analysis of the importance of single predictors for the regression task^34^. Statistical significance of the *R*^*2*^ values as well as of predictor importance was determined using a modified t-test that takes the sample dependence due to the CV into account^38^.

#### Step 2: Assessing the significant art attributes predicting beauty separately for each culture group

Second, we assessed which art attributes significantly contribute to the prediction of the beauty ratings using data from native Japanese (Step 2.1.) or German groups (Step 2.2) separately. By building an identical model for Japanese and German native groups to predict beauty ratings, we aimed to understand what the significant contributors to the prediction of the beauty ratings are for each group. We note that all procedures for building a predictive regression model for the beauty rating scores, as well as evaluating the model, were identical to building the models in Step 1. However, the predictor representing the mother tongue of the art viewers was excluded since only the data from native Japanese or German groups were included in the models in this step. Thus, only the 17 art attributes were included as predictors in Steps 2.1 and 2.2.

#### Step 3: Assessing if there are differences in the art attributes predicting beauty between two culture groups

To detect cultural differences, we compared the two models built in Steps 2.1 and 2.2 and examined if the contribution to predict the beauty of each predictor significantly differed between the two culture groups. The statistical comparison of the feature importance was carried out using a modified *t*-test that takes the sample dependence due to the CV into account^38^. T-test results where Bonferroni corrected for multiple comparisons. Figure 3 presents differences in the contributions of the 17 predictors to the beauty judgments between native Japanese (Step 2.1) and German (Step 2.2) speakers.

### Significance statement

In our research we explore beauty judgments in visual art and investigate which art attributes might contribute to this judgment considering diverse cultural backgrounds. We analyzed how Japanese and German speakers rated artworks, discovering that both groups agree on certain aspects, such as visual harmony and positive valence, but differ significantly in others. For instance, the native Japanese speakers prefer simplicity, while the native German speakers favor complexity. This highlights how deeply our cultural roots shape our appreciation of art, underscoring the importance of considering cultural diversity in understanding the appeal of beauty in visual art.

## Results

In this section, we present the results corresponding to the analysis steps 1–3 described in the sub-section: Machine learning based data analysis approach.

### Step 1: Assessing the Significance of the Culture group to Predict Beauty

The result of the *t*-test for Step 1 is shown in Table 2. The average *R*^*2*^ value of the predictive model was 0.22, meaning that the model explains 22% of the variance in the beauty rating scores with the provided predictors. The average *R*^*2*^ value of 0.22 is statistically significantly higher than the average *R*^*2*^ value obtained with shuffle data models (*p* < 0.0001). A correlation heatmap between all measures, as well as the results of Principle component analysis (PCA) for the model, can be found in Supplementary Information (Figs. S1, S2, respectively).

**Table 2:** The results of model predictions in distinct phases.

Models	*R*^*2*^ original mean (*SD*)	*R*^*2*^ shuffle mean (*SD*)	*p* value of difference for the *R*^*2*^ shuffle mean
Step 1	0.22 (0.03)	− 0.02 (0.02)	*p* < .0001
Step 2.1 Japanese	0.22 (0.04)	− 0.03 (0.03)	*p* < .0001
Step 2.2 German	0.21 (0.04)	− 0.02 (0.02)	*p* < .0001

**Figure 1:**
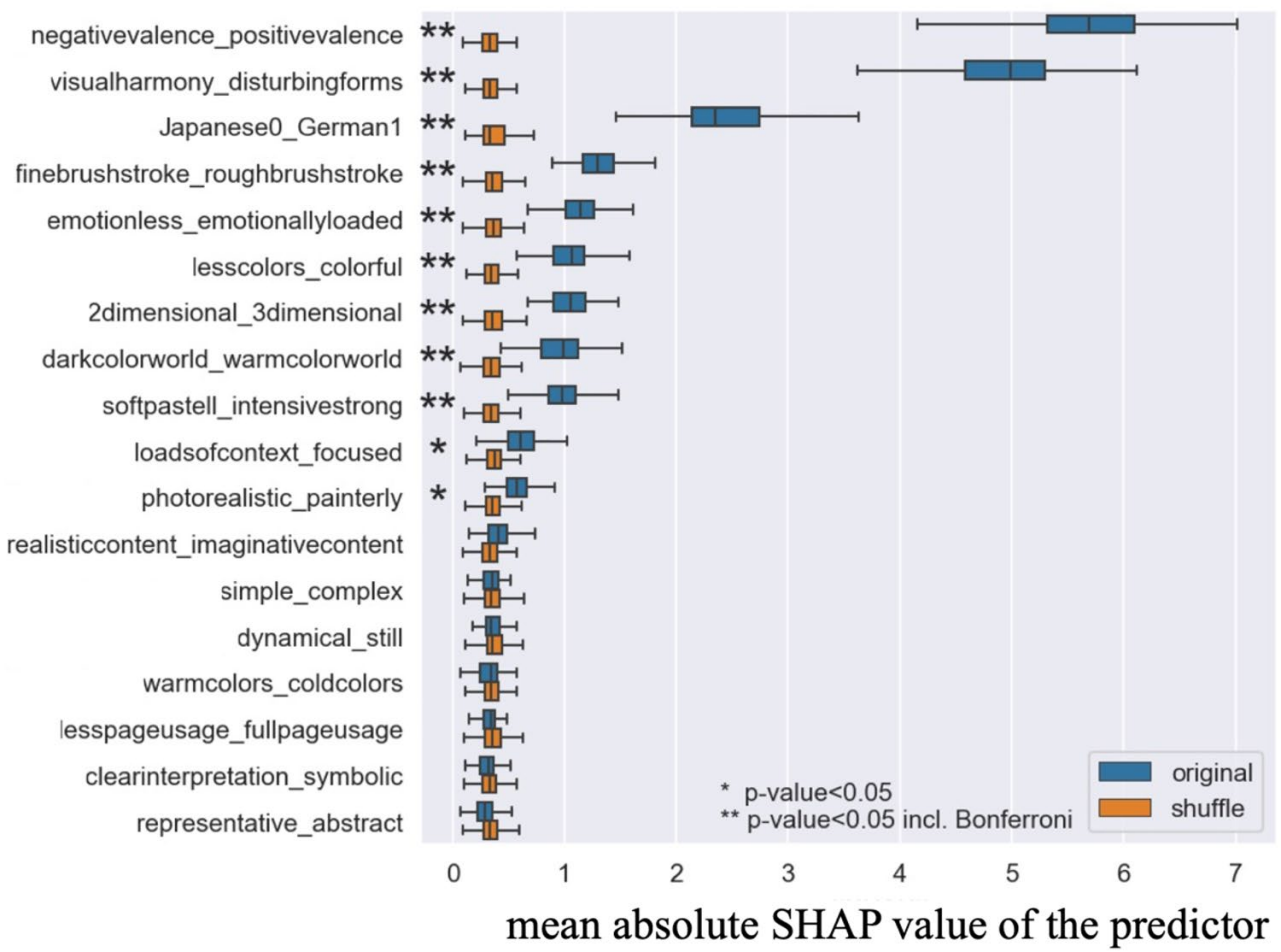
The mean absolute SHAP values for all 18 predictors in the model in Step 1, including 17 art attributes and the native language of the participants. The predictor in the figure is sorted from the top to the bottom, based on the mean absolute SHAP value of the predictor. Hence, the predictor, shown on the top, is the most important predictor for beauty judgments. [*represents the uncorrected *p*-value of a modified *t*-test; **represents the corrected *p*-value with Bonferroni correction].

Figure 1 presents the contributions of the 18 predictors to the beauty ratings. The total impact of each predictor was measured via the mean of the absolute SHAP values. As in the previous section, SHAP value represents the importance of a predictor. Specifically, they provide information on how much the prediction changes given a specific predictor value. The average absolute SHAP value of a predictor gives how much the prediction is changed by this predictor on average. Importantly, the predictor, the native language of the art viewers (Japanese 0, German 1), was significantly important for predicting beauty rating scores compared with SHAP results from shuffle data models. Accordingly, it seems plausible to separate the data of native Japanese and German speakers (Step 2) to see how the importance of predictors differs between the two culture groups (Step 3).

#### Step 2.1 Assessing the Significant Attributes Predicting Beauty: Japanese Group

**Figure 2:**
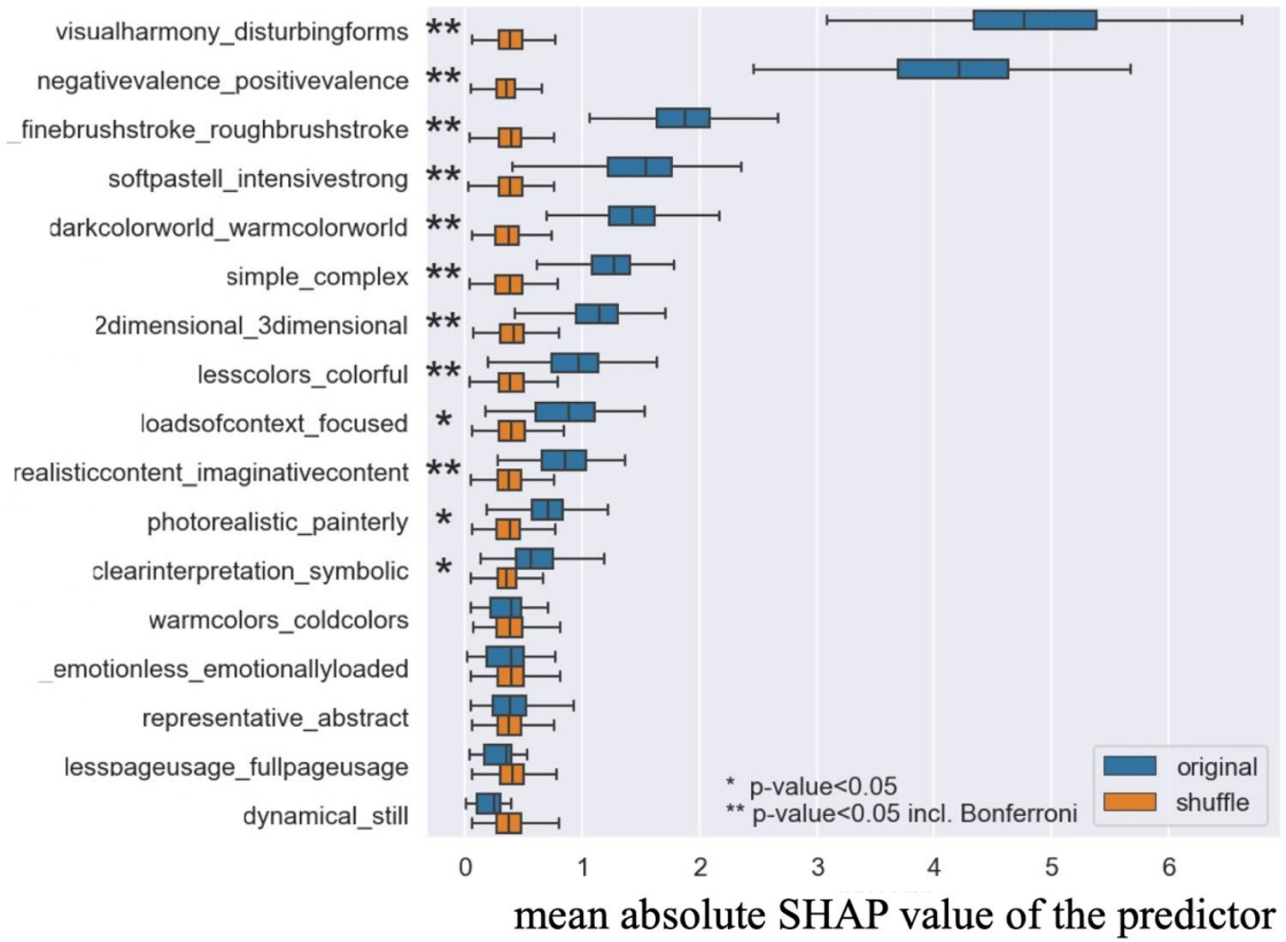
The mean absolute SHAP values for all 17 art attributes in the model in Step 2.1 using only the data from native Japanese group.

The result of the predictive model in Step 2.1 is shown in Table 2. The average *R*^*2*^ value of the predictive model was 0.22, meaning that the model could explain 22% of the variance in the beauty rating scores. Further, Fig. 2 presents the contributions of the 17 predictors to the beauty judgments made by native Japanese participants. As in Step 1, the impact of each predictor was measured via the mean absolute value of SHAP values. A correlation heatmap between all measures, as well as the results of PCA for the model, can be found in Supplementary Information (Figs. S3, S4, respectively).

In Fig. 2, the predictors are listed by their importance in predicting beauty ratings. For example, *visualharmony_disturbingforms* was the most important feature for predicting beauty, and the second most important feature was *negativevalence_positivevalence*.

The results showed that nine predictors were significant in predicting beauty rating scores from native Japanese group: *visualharmony_disturbingforms,* *negativevalence_positivevalence, finebrushstroke_roughbrushstroke, softpastell_intensivestrong, darkcolorworld_warmcolorworld, simple_complex, 2dimensional_3dimensional, lesscolors_colorful, realisticcontent_imaginativecontent.* Note that we only present the significant results with Bonferroni correction here to discuss the results. The directions of the results are shown in Table 3.

**Table 3:** The directions of the results in the model in Step 2.1.

Significant predictor	Direction of results
Formal perceptual attributes	
visualharmony_disturbingforms	Paintings with high visual harmony were evaluated as more beautiful, and with disturbing form as less beautiful.
finebrushstroke_roughbrushstroke	Paintings with fine brush stroke were evaluated as more beautiful, and with rough brush stroke as less beautiful.
softpastell_intensivestrong	Paintings with soft pastel color were evaluated as more beautiful, and with intensive strong color as less beautiful.
darkcolorworld_warmcolorworld	Paintings with warm color world were evaluated as more beautiful, and with dark color world as less beautiful.
simple_complex	Simple paintings were evaluated as more beautiful, and complex ones as less beautiful.
2dimensional_3dimensional	3 dimensional paintings were evaluated as more beautiful, 2 dimensional ones as less beautiful.
lesscolor_colorful	Colorful paintings were evaluated as more beautiful, and ones with less colors as less beautiful.
Content representational attributes	
negativevalence_positivevalence	Paintings with positive valence were evaluated as more beautiful, and with negative valence as less beautiful.
realisticcontent_imaginativecontent	Realistic paintings were evaluated as more beautiful, and imaginary ones as less beautiful.

As the absolute SHAP values represent the importance of each predictor, it does not tell the directionality of the results. For example, whether the participants evaluate high in harmony or disturbing forms of the paintings as more beautiful cannot be seen in the absolute SHAP value. The interpretation of the result direction was achieved by seeing a plot, showing the exact SHAP values and feature values together. The plot for Step 2.1 can be found in Supplementary Information, Figure S5, and the one for Step 2.2. can be found also in Supplementary Information, Figure S8.

#### Step 2.2 Assessing the significant art attributes predicting beauty: German group

**Figure 3:**
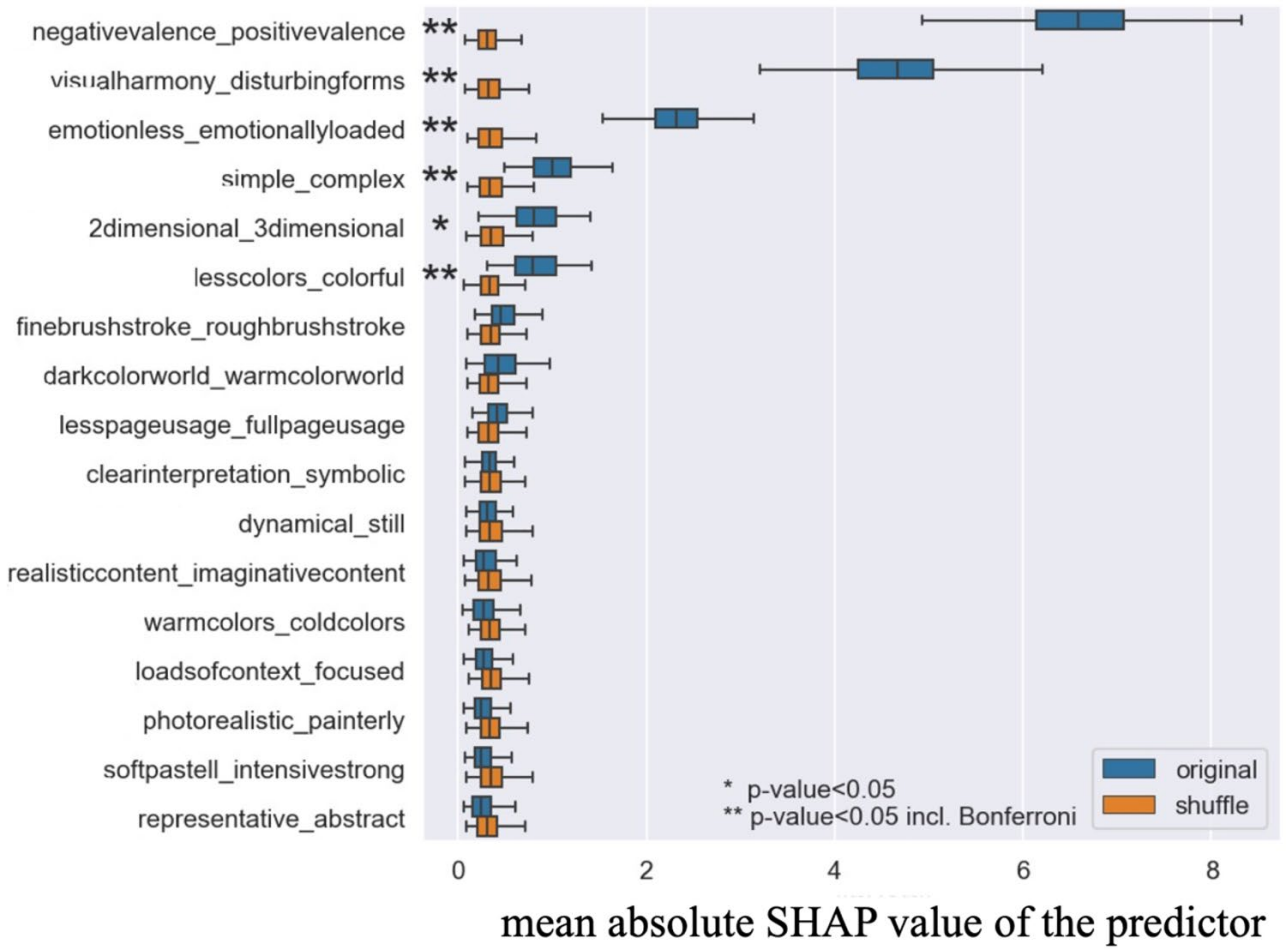
The mean absolute SHAP values for all 17 art attributes in the model in Step 2.2 using only the data from the native European group.

The result of the predictive model in step 2.2 is shown in Table 2. The average *R*^*2*^ value of the predictive model was 0.21, meaning that the model could explain 21% of the variance in the beauty rating scores. Further, Fig. 3 presents the contributions of the 17 predictors to the beauty judgments made by the European group. As in Steps 1 and 2.1, the impact of each predictor was measured via the mean absolute value of SHAP. A correlation heatmap between all measures, as well as the results of PCA for the model, can be found in Supplementary Information (Figs. S6, S7, respectively).

The results showed that five predictors were statistically significantly important to predict beauty rating scores, namely, *negativevalence_positivevalence, visualharmony_disturbingforms, emotionless_emorionallyloaded, simple_complex, lesscolors_colorful.* Again, we only present the significant results with Bonferroni correction here to discuss the results. The directions of the results are shown in Table 4.

**Table 4:** The directions of the results in the model in Step 2.2.

Significant predictors	The direction of the results
Formal perceptual attributes	
visualharmony_disturbingforms	Paintings with visual harmony were evaluated as more beautiful, and with disturbing forms as less beautiful.
simple_complex	Complex paintings were evaluated as more beautiful, and simple ones as less beautiful
lesscolor_colorful	Colorful paintings were evaluated as more beautiful, and paintings with less colors as less beautiful.
Content representational attributes	
negativevalence_positivevalence	Paintings with positive valence were evaluated as more beautiful, and with negative valence as less beautiful.
emotionless_emotionloaded	Emotion loaded paintings were evaluated as more beautiful, and emotion less ones as less beautiful.

#### Step 3. Assessing differences in art attributes predicting beauty between two culture groups

**Figure 4:**
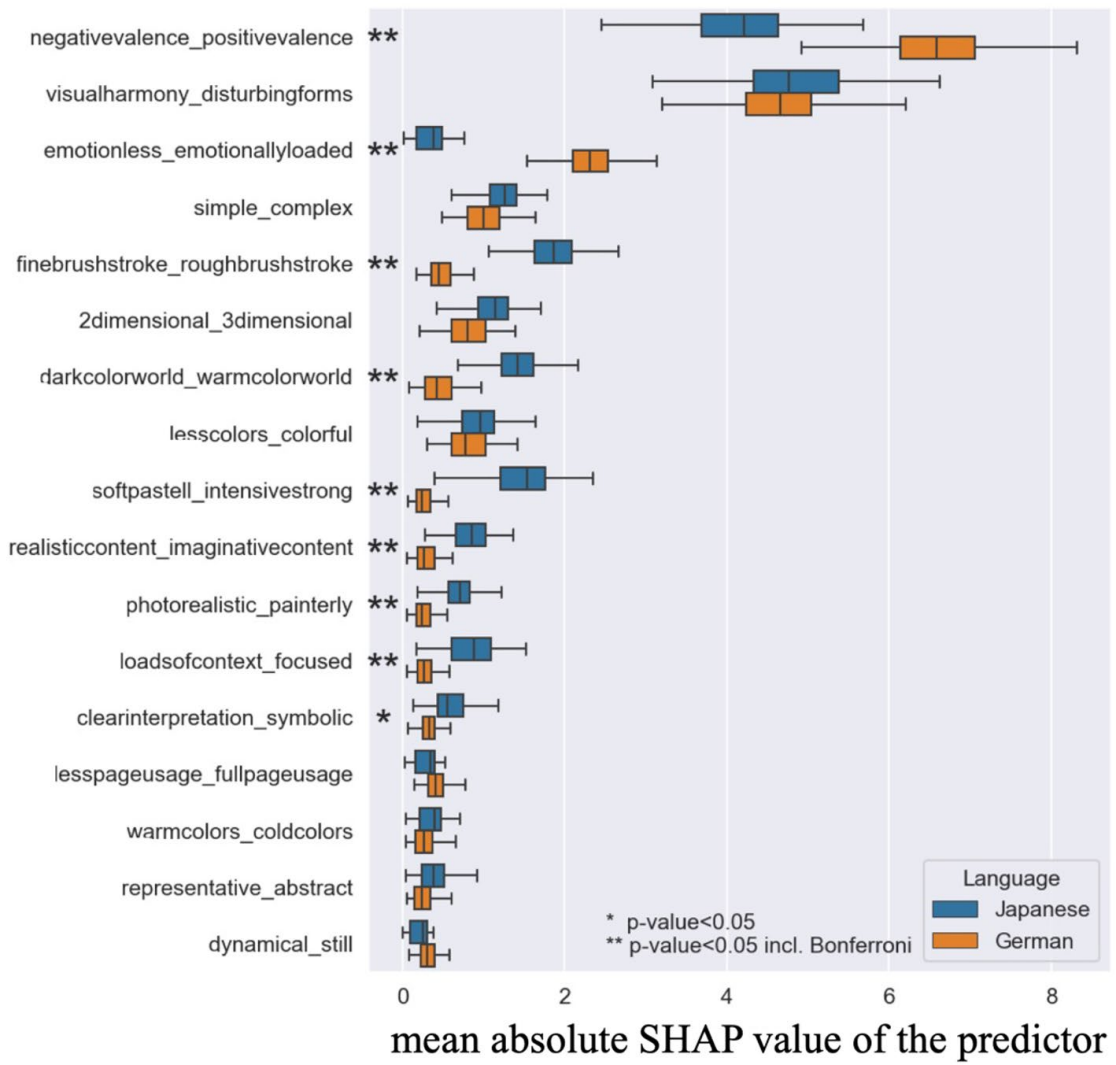
The results of the differences in the contributions of the 17 predictors to the beauty judgments between native Japanese (Step 2.1) and German (Step 2.2) speakers.

The statistical comparison of the feature importance between the two models built in Steps 2.1 and 2.2 was carried out using a modified *t*-test that takes the sample dependence due to the CV into account^38^. *t*-test results where Bonferroni corrected for multiple comparisons. Figure 4 presents differences in the contributions of the 17 predictors to the beauty judgments between two culture groups.

The results showed that there were significant differences between two groups in the importance of eight predictors: *negativevalence_positivevalence, emotionless_emorionallyloaded, finebrushstroke_roughbrushstroke, darkcolorworld_warmcolorworld, softpastell_intensivestrong, realisticcontent_imaginativecontent, photorealistic_painterly, loadofcontext_focus.* Again, we only present the significant results with Bonferroni correction here to discuss the results. How the results differed for the eight variables is described in Table 5.

**Table 5:** The directions of the cross-cultural differences.

Predictors	Results
Formal perceptual attributes	
finebrushstroke_roughbrushstroke	While this feature was important for Japanese people, it was not for German people: Japanese evaluate more fine strokes as more beautiful.
darkcolorworld_warmcolorworld	While this feature was important for Japanese people, it was not for German people: Japanese evaluate warm color as more beautiful.
softpastell_intensivestrong	While this feature was important for Japanese people, it was not for German people: Japanese evaluate pastel color as more beautiful.
Content represnetational attributes	
negativevalence_positivevalence	This feature was important for both cultures, but more important for German speaking people: same direction, always the positive was appraised as more beautiful.
emotionless_emorionallyloaded	While this feature was important for German, it was not for Japanese people: German evaluated more emotionally loaded paintings as more beautiful.
realisticcontent_imaginativecontent	While this feature was important for Japanese people, it was not for German people: Japanese evaluate realistic painting as more beautiful.
photorealistic_painterly	While this feature was more important for Japanese people than for German people: The Japanese people evaluate photorealistic painting as more beautiful. Note this feature was not significant in the Step 2.1.
loadofcontext_focus	While this feature was more important for Japanese people than for German people: The Japanese people evaluate paintings with loads of contents as more beautiful﻿. Note this feature was not significant in the Step 2.1.

## Discussion

In the present study, we explored art attributes that contribute to the judgment of beauty in Western visual artworks. We investigated whether these attributes exhibit variations across raters with different cultural backgrounds, as operationalized by their mother tongue. Our study aimed to address two research questions:

1. Which attributes in artworks contribute to the judgment of beauty?
2. Does cultural background, operationalized as the difference between Japanese and German native speakers, significantly affect beauty judgments? Moreover, if so, what are the intercultural differences and commonalities in the art attributes that predict beauty?

In the following discussion, we first walk through the cross-cultural commonalities and the differences in art attributes predicting beauty judgments between the two culture cohorts. The results will also be discussed in relation to the cognitive stages involved in aesthetic judgments. Finally, we note the limitations and the future potential of this line of research.

### Common art attributes determining beauty judgments

Our analysis revealed that four art attributes consistently influenced beauty judgments across all participants in both groups, Japanese and German speakers. These attributes were *visual harmony, color variety, valence,* and *complexity.*

Irrespective of native language, participants tended to evaluate paintings with higher visual harmony, more color, and positive valence as more beautiful. Conversely, artworks with disturbing forms, fewer colors, and negative valence were perceived as less beautiful. The identification of visual harmony and the positive valence as cross-cultural commonalities of beauty align with historical and contemporary aesthetic discussions^39–44^. However, note that the results of Step 3 revealed that although the attribute *valence* was an important factor in predicting beauty for both groups, the importance of this attribute was significantly larger for the German-speaking group, showing that the level of the importance differed across the two cultural cohorts.

Interestingly, the attribute *complexity* also emerged as a consistent predictor of beauty across cultures. However, Japanese and German groups showed opposing preferences within this attribute. While the Japanese group favored simpler paintings, the German group found more complex artworks to be beautiful. This intercultural difference challenges some established notions in empirical aesthetics, which have often suggested a preference for medium to high levels of complexity independent of cultural heritage^6,10,11,45^. However, our results open a new debate on complexity levels: the preference for simplicity among the Japanese group may reflect cultural values related to Zen principles, emphasizing simplicity and quietness. As can be seen in terms of Zen principles such as *Kisha* (喜捨)—meaning discard with pleasure, being and living simply, mentally and physically—simplicity is appreciated in East Asian cultures. Consequently, appreciation for greater simplicity is also elicited in preference for artworks. In Japanese as well as Chinese landscapes, it is quite a common aesthetic standard to utilize void spaces, which might make the space seem simpler, or at least reduce the visual complexity^21^. Further, simplicity, as well as seen and experienced quietness, is intentionally implemented in the design of Japanese traditional gardens^46^. Nevertheless, in our study design, as we only employed Western art paintings, this finding underscores the potential impact of cultural upbringing on aesthetic evaluations of paintings.

Of the four art attributes significantly predicting beauty judgments in both cultural cohorts, three art attributes belong to the formal perceptual attributes. In AAA, these attributes correspond to early/intermediate human vision^7,8,14^. Our results suggest that how one judges beauty in visual art might already be determined in the early stages of information processing, e.g., when we process the perceptual qualities of the stimuli or object classification.

### Inter-cultural variations in art attributes influencing beauty

Our results from Step 3 revealed that seven art attributes were important predictors of beauty for one cultural group but not the other. For the German group, *emotional expressiveness* was a key attribute influencing beauty judgments, whereas the same significance did not hold for the Japanese group. In contrast, *brushwork, color world, saturation,* and *realism/imaginativeness* were influential only for the Japanese group. Additionally, though the results of Step 2.1 did not show a significant impact on predicting beauty, *accurate object representation* and *focus* were significantly more important for the Japanese compared to the German group. Of the seven art attributes showing inter-cultural variations in influencing beauty judgments, three attributes (*brushwork, color world, saturation)* belong to the formal perceptual attributes, while the other four attributes (*emotional expressiveness, realism/imaginativeness, accurate object representation, focus*) belong to the content representational attributes. Hence, socio-cultural factors seem to influence beauty judgments not only in earlier/intermediate cognitive processing stages but also in later stages. This makes sense, considering that cross-cultural differences can be found not only in what type of visual stimuli one might more frequently encounter but also in what is shared and reinforced in the members of a society^47–49^.

For instance, the importance of *brushstroke* for the Japanese group may reflect cultural writing practices such as writing *Kanjis*, where brushstroke quality is critical to creating beautiful characters. Similarly, *Shodo* (書道) or *Shuuji* (習字), which is a form of calligraphy, is one of the popular art forms in Japan and is also in the curriculum in junior/high school. In Shodo and Shuuji, ink is used with a thick brush to write Japanese words, i.e., Kanjis, on thin traditional Japanese paper. Hence, considering that the evaluation for brushstrokes is commonly implemented in everyday life for the Japanese people, it seems plausible that this attribute was more important to the Japanese group but not to the German group, who do not have such traditions.

The importance of *emotional expressiveness* for German speakers could be related to cultural differences in expressing emotions in daily life and during social interactions. Europeans, on average, are often described as more expressive, while Japanese cultures place high value on restraint, quietness, and formality^21,50,51^. These differences in emotional expression align with the social norms and practices observed in each culture. Certainly, these results somehow go along with the cliché of the two cultures, which warrants further study of the cultural practices and norms in combination with visual art rating studies to challenge or elucidate the origins of these findings.

Furthermore, the attributes *realism/imaginativeness* and *accurate object representation* align with prior research, suggesting that cultural values and practices can shape aesthetic preferences. For instance, studies have shown that Western individuals value uniqueness, while East Asians often prefer representations of conformity. Kim and colleagues^52^ reported that while North American people show their preference for unique objects, e.g., an uncommon pen, East Asians prefer something representing conformity, e.g., a common pen. Our findings that Japanese participants evaluated realistic and accurate depictions as more beautiful align with this pattern. This suggests that cultural values and reinforcement cycles within cultures can shape aesthetic preferences.

We also found interesting results considering color: while for the German group, only the *color variety* significantly contributed to the beauty judgment, for the Japanese group, beyond *color variety*, other color features were also significantly relevant, such as *color saturation*, *color temperature*, and *color world*. Ishii et al.^53^ found a similar tendency in the colorings of patterns, with participants preferring their own-culture colorings more than those from different cultures^55^. They discussed that children in Japan and Canada gave feedback about the children’s colorings that were consistent with their culture’s values. Our results suspect that despite color variety (see also studies on ferocious colors^30,54^ and cross-cultural studies on the affective meanings of color^55–57^) color might be more important for Japanese than for German populations; not necessarily about color-associations in daily life, but in artworks.

Finally, the attribute *focus* reflects different ways of visual exploration in Eastern and Western Cultures^21,58^: while East Asian individuals tend to focus on contextual information in the visual field, Western individuals typically prioritize focal objects. Our results indicate that Japanese speakers evaluated paintings with more contextual information as more beautiful, mirroring the cultural differences observed in visual exploration strategies.

### Limitations

Firstly, it is important to acknowledge that our list of attributes does not exhaustively encapsulate beauty judgments. Nevertheless, we focused on a core set of widely recognized attributes, incorporating cross-cultural elements to ensure the list was comprehensive yet manageable considering participants’ fatigue and model integration (see for further detailed discussion, prior research^17,18^). This approach was chosen to avoid overwhelming participants and maintain the efficacy of the prediction models by keeping the number of factors at a reasonable level. Hence, based on our results, we hope that future studies will include other attribute scales to further advance this line of research using a data-driven approach.

One further major limitation was the difference in data collection due to the Covid-19 pandemic restrictions during the Japanese data collection period. We, therefore, could not control potential differences due to the image presentation itself.

The research focused on a specific subset of Western and East Asian cultures, represented by Japanese- and German- native speaking participants. Consequently, the findings may not be generalizable to all Western or Asian cultures. Additionally, the artworks used in our stimulus sample were exclusively Western art images. We, therefore, cannot generalize to other forms of art. We used a large set of 54 artworks selected from our databank^31^, differing on various pre-rated dimensions rather than a higher cultural variety^59^. However, given our assumption that beauty arises from a set of cognitive processes from any stimuli, we suppose the same mechanisms could work when evaluating a beautiful non-artistic object (e.g., an object of everyday life). Future studies could extend our design to artworks with higher diversity, i.e., including the artworks from East Asia, or to include stimuli other than paintings, to identify further communalities and disparities in art attributes regarding beauty judgments among populations with significant cultural differences.

We aimed to approach beauty from a different perspective adding value to the contemporary theories of cognitive aesthetics^60–62^. Remarkably, we have not aimed to define *what* beauty is. Further, our purpose was explicitly not to tackle beauty from a philosophical and theoretical perspective. Instead, we understand the judgment of beauty as a non-monolithic and varying cluster of cognitive processes shaped by personal experience and socio-cultural influences. Our studies focused, therefore, on a behavioral assessment of expressing one’s own experience of finding a visual artwork beautiful. Nevertheless, our approach could nurture and benefit from further debates in the philosophy of aesthetics or sociology^63–66^.

## Conclusion

Our study has offered insights into the intricate relationship between cultural backgrounds and the evaluation of beauty in visual artworks. On the one hand, we found that specific attributes consistently played a role in predicting beauty judgments across two cultural contexts—German and Japanese-speaking samples. On the other hand, we identified intercultural variations in attribute predictions, emphasizing the crucial role of culture in influencing art experiences, evaluations, and cognitive processing^7,8,14,49,67^.

Our application of data-driven machine learning regression models, complemented by permutation importance analysis, has proven to be a robust approach^17,18^. It has allowed us to delve deeper into the relevance of individually evaluated art attributes in predicting beauty judgments. This methodological approach not only advances our understanding of culture’s influence on art appreciation but also paves the way to future explorations of the interplay between culture and aesthetics using data-driven analysis instead of and next to hypothesis-driven approaches^24,35^.

In addition to presenting which attributes contribute to the judgment of beauty of visual artworks and highlighting intercultural differences and commonalities, we found, our research provides an answer to a primary question: *“Yes, diverse cultural backgrounds significantly impact the prediction of beauty judgments.”* Yet, this conclusion is just the beginning of a more profound exploration of the complex dynamics between culture and aesthetics.

## Data availability

The datasets generated and/or analyzed during the current study are available in the OSF repository: https://osf.io/x7jkc/?view_only=0b49c691b4624e499bc9534e06eaa63f.

### Supplementary Information

Supplementary Information.
