# Supplementary material for: Cross-cultural comparison of beauty judgments in visual art using machine learning analysis of art attribute predictors among Japanese and German speakers
Source: Sci Rep. 2024 Jul 10;14:15948. doi: 10.1038/s41598-024-65088-z (PMC11237067; doi:10.1038/s41598-024-65088-z)
Supplement: Supplementary file 1 — Supplementary Information. [file 41598_2024_65088_MOESM1_ESM.docx]

**Supplementary Information for**

**Cross-Cultural Comparison of Beauty Judgments in Visual Art Using Machine Learning Analysis of Art Attribute Predictors Among Japanese and German Speakers**

Jan Mikuni^1*‡^, Blanca T.M. Spee^1,2,3*‡^, Gaia Forlani^2,4‡^, Helmut Leder^1,3^, Frank Scharnowski^1, 3^, Koyo Nakamura^3^, Katsumi Watanabe^5^, Hideaki Kawabata^6^, Matthew Pelowski^1,3^, David Steyrl^1,3^

1 Vienna Cognitive Science Hub, University of Vienna, Vienna, Austria

2 Radboud University Medical Centre; Donders Institute for Brain, Cognition and Behavior; Department of Neurology; Center of Expertise for Parkinson & Movement Disorders; Nijmegen, The Netherlands

3 Department of Cognition, Emotion, and Methods in Psychology, Faculty of Psychology, University of Vienna, Vienna, Austria

4 Radboud University Medical Centre; Donders Institute for Brain, Cognition and Behavior; Department of Rehabilitation; Center of Expertise for Parkinson & Movement Disorders; Nijmegen, The Netherlands

5 Faculty of Science and Engineering, Waseda University, Tokyo, Japan

6 Department of Psychology, Faculty of Letters, Keio University, Tokyo, Japan

‡J.M., BTM.S., and G. F. contributed equally to this work and share the first authorship.

*Correspondence concerning this article should be addressed to

Jan Mikuni, Vienna Cognitive Science Hub, University of Vienna, Kolingasse 14-16, Vienna 1090, Austria. E-mail: jan.mikuni@univie.ac.at, and Blanca T.M. Spee, Vienna Cognitive Science Hub, University of Vienna, Vienna, Austria; Radboud University Medical Centre; Donders Institute for Brain, Cognition and Behavior; Department of Neurology; Center of Expertise for Parkinson & Movement Disorders; Nijmegen, The Netherlands, e-mail: blanca.spee@univie.ac.at

**Supplementary Information Figures**


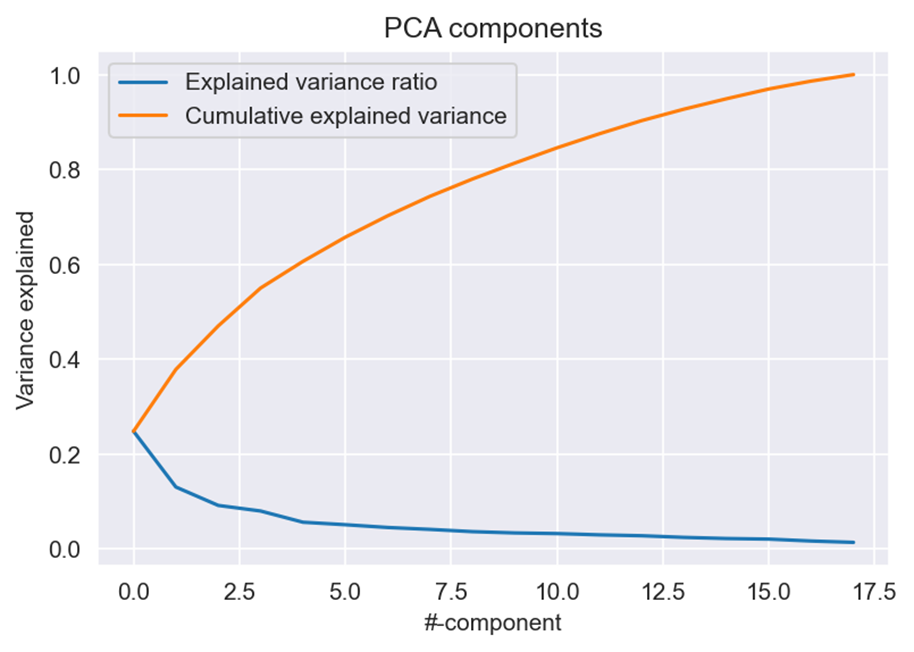


**Figure S1.** Principal component analysis for Step 1. Hence, data from both native Japanese and native German speakers are included. This figure presents the results of the PCA, showing the percentage of the total variance explained by each principal component; all but one of the components were necessary to account for over 99% of the total variance in the data set; this suggests that reducing the data to a lower-dimensional space would result in significant information loss.


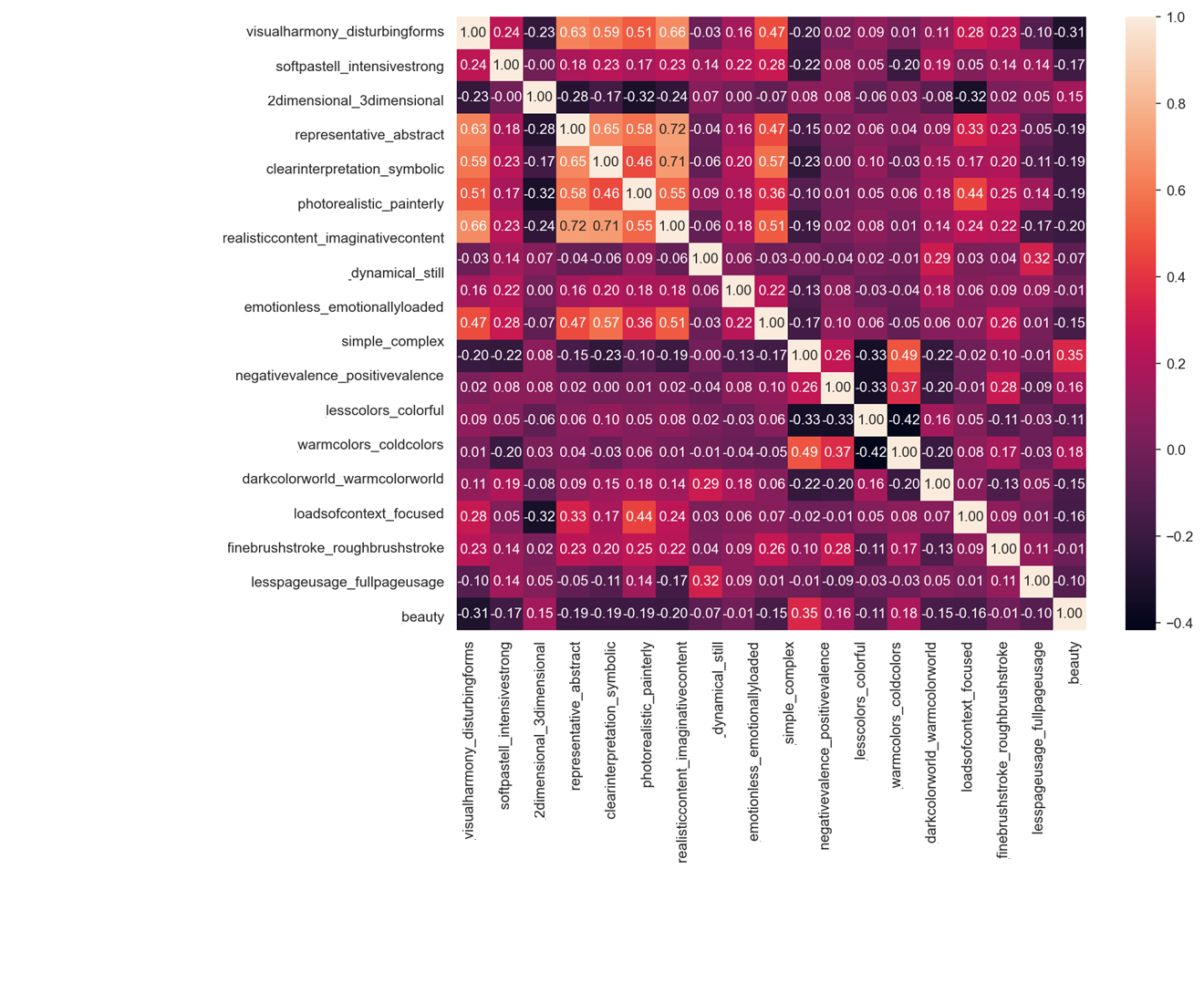


**Figure S2.** Exhaustive heatmap of correlations between art attributes and beauty judgment, using the data from both native Japanese and native German participants. Statistical significance was not assessed. Although strong correlations between predictors are observed for some cases, they do not highlight a strong correlation with beauty. Despite some predictors showing interdependence (historically expected), our method accommodates this, as the coefficients do not reach 1, ensuring each predictor still carries unique information.


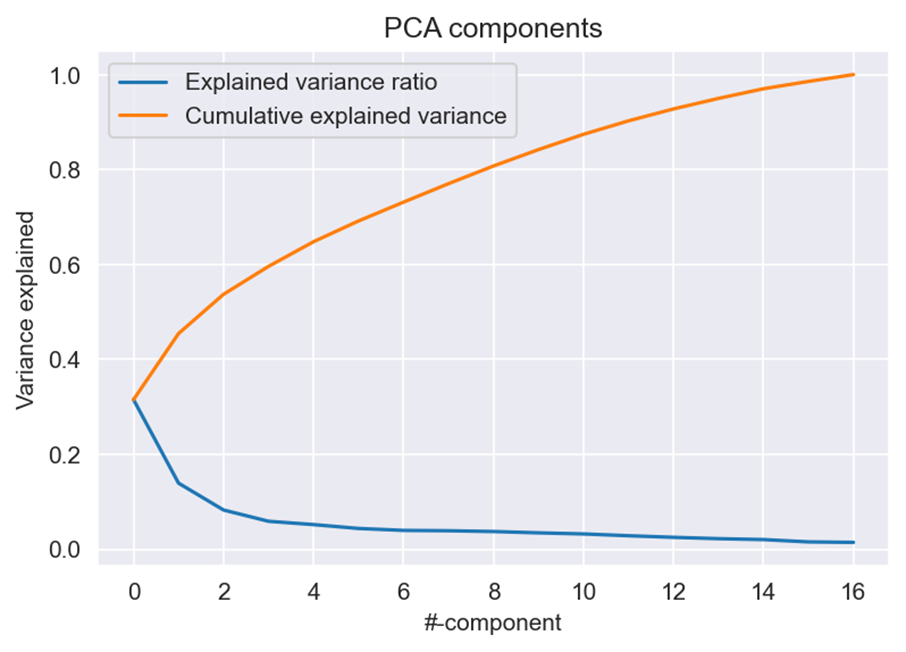


**Figure S3.** Principal component analysis for Step 2.1. Hence, data from native Japanese speakers are included. This figure presents the results of the PCA, showing the percentage of the total variance explained by each principal component; all but one of the components were necessary to account for over 99% of the total variance in the data set; this suggests that reducing the data to a lower-dimensional space would result in significant information loss.


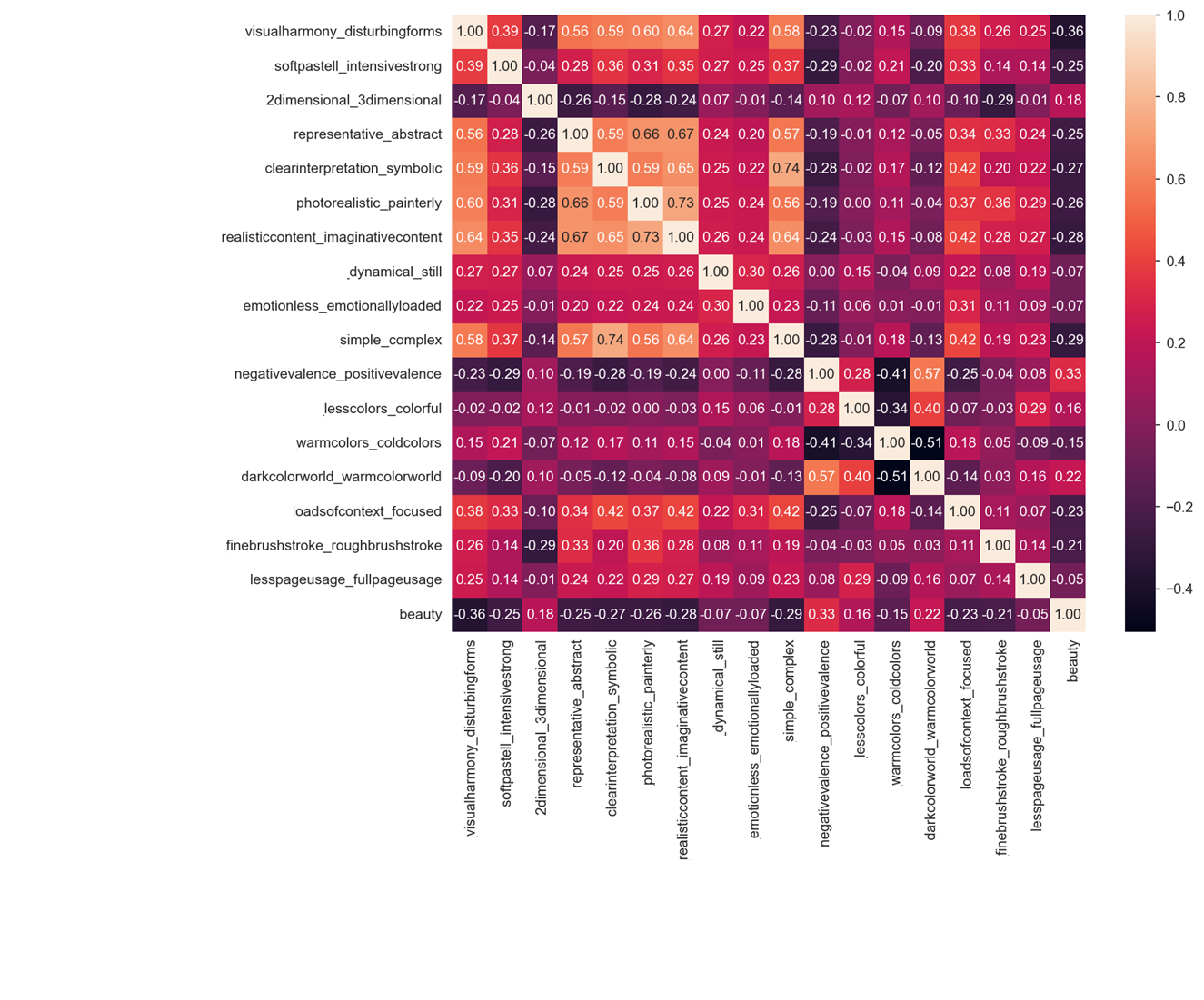


**Figure S4.** Exhaustive heatmap of correlations between art attributes and beauty judgment, using the data from native Japanese participants. Statistical significance was not assessed. Although strong correlations between predictors are observed for some cases, they do not highlight a strong correlation with beauty. Despite some predictors showing interdependence (historically expected), our method accommodates this, as the coefficients do not reach 1, ensuring each predictor still carries unique information.


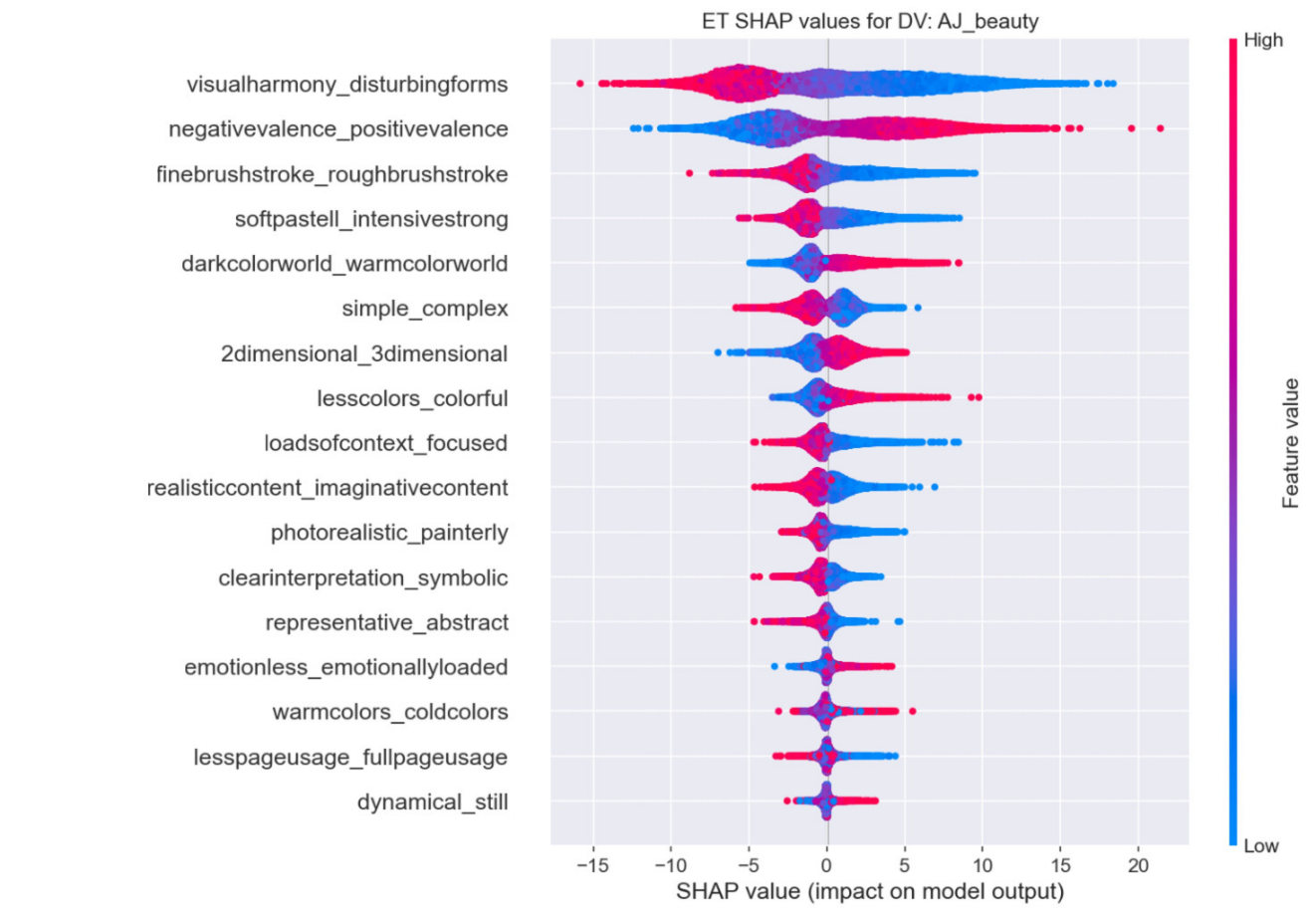


**Figure S5.** A violin plot showing the SHAP values of each variable, predicting beauty, with feature values for Step 2.1, using the data from native Japanese speakers. Taking the first variable “visualharmony_disturbingforms” as an example, the figure shows that the Japanese speakers evaluated the visual artworks, which are high in visual harmony, as more beautiful.


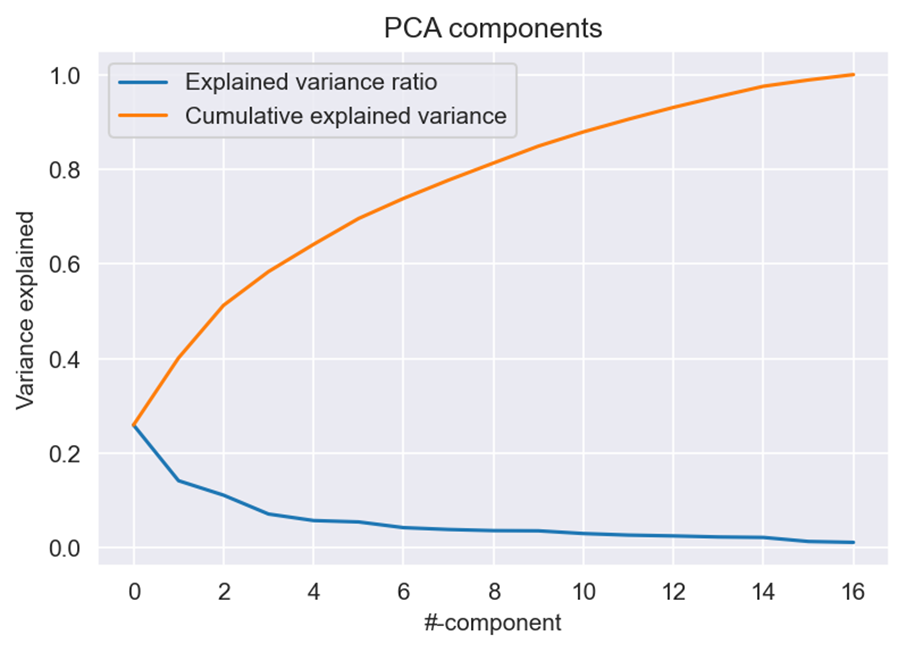


**Figure S6.** Principal component analysis for Step 2.2. Hence, data from native German speakers are included. This figure presents the results of the PCA, showing the percentage of the total variance explained by each principal component; all but one of the components were necessary to account for over 99% of the total variance in the data set; this suggests that reducing the data to a lower-dimensional space would result in significant information loss.


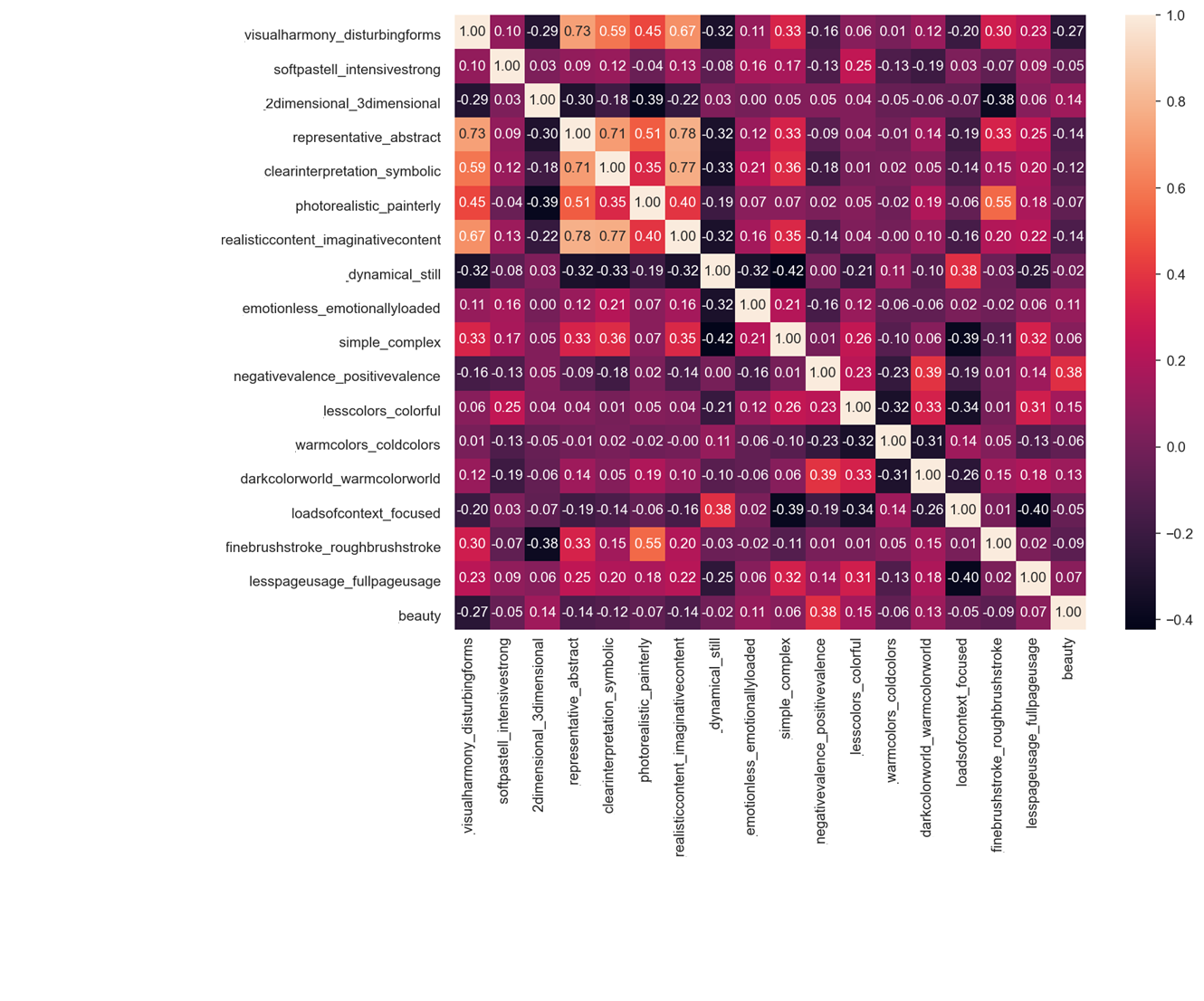


**Figure S7.** Exhaustive heatmap of correlations between art attributes and beauty judgment, using the data from native German participants. Statistical significance was not assessed. Although strong correlations between predictors are observed for some cases, they do not highlight a strong correlation with beauty. Despite some predictors showing interdependence (historically expected), our method accommodates this, as the coefficients do not reach 1, ensuring each predictor still carries unique information.


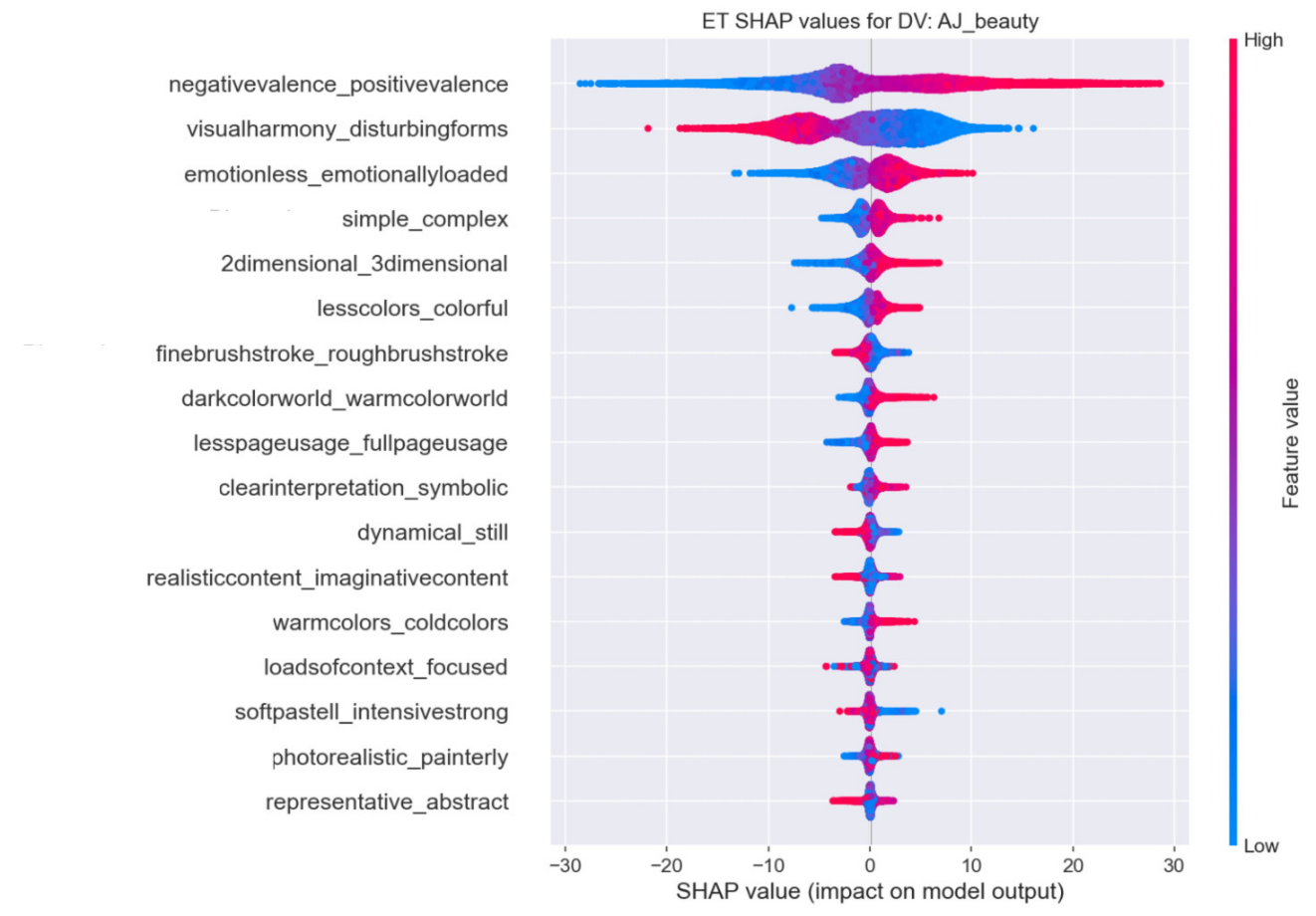


**Figure S8.** A violin plot showing the SHAP values of each variable, predicting beauty, with feature values for Step 2.2, using the data from native German speakers.

**Supplementary Information Tables**

**Table S1.** Items of art-attributes along their semantic differential dimension poles (independent variables used in machine learning analysis). German version.

|  | **Anweisung:** *Wie schön finden Sie das Kunstwerk?* | *Bitte bewerten Sie das Kunstwerk anhand der unterschiedlichen Attribute:* | |
| --- | --- | --- | --- |
| **Attribute** | **Items** | **Negativer Pol (Minimum)** | **Positiver Pol (Maximum)** |
| **i. Formal-perzeptive Attribute** | a. Visuelle Harmonie (Balance) | visuelle Harmonie, proportional | eigenartig, seltsame Formen |
|  | b. Tiefenwahrnehmung | zwei-dimensional | drei-dimensional |
|  | c. Komplexität | einfach | komplex |
|  | d. Farbsättigung | sanft, pastell | intensive, kräftig |
|  | e. Farbvielfalt | wenige Farben | Farbvielfalt |
|  | f. Farbtemperatur | warme Farben | kalte Farben |
|  | g. Farbwelt | dunkle Farbwelt | helle Farbwelt |
|  | h. Pinselführung | feine Pinselführung | grobe Pinselführung |
|  | i. Ausnützung der Zeichenfläche | wenig Ausnützung der Malfläche | sämtliche Ausnützung der Malfläche |
| **ii. Inhaltlich-repräsentative Attribute** | j. Abstraktion | repräsentativ | abstrakt |
|  | k. Imagination | realistischer/s Inhalt/ Thema | imaginär, unwirklich, fantastisch |
|  | l. Symbolismus (Ambiguität) | eindeutig (klare Interpretation der Darstellung) | symbolisch (mehr Interpretationsfreiraum) |
|  | m. Akkurate Objektdarstellung | fotorealistisch | malerisch |
|  | n. Lebendigkeit, Animation | dynamisch | still |
|  | o. Emotionalität | emotionslos | emotional aufgeladen |
|  | p. Valenz | negative Stimmung | positive Stimmung |
|  | q. Fokussierung | viel Kontext/Umgebung  im Bild | fokussierter Inhalt |

**Table S2.** Items of art-attributes along their semantic differential dimension poles (independent variables used in machine learning analysis). Japanese version.

|  | **Instructions:** この芸術作品は美しいですか？ | それぞれの次元・特性ごとに芸術作品を評価してください： | |
| --- | --- | --- | --- |
| **Attribute** | **Items** | 全く当てはまらない(Minimum) | 非常に当てはまる(Maximum) |
| **i. 形式的知覚属性** | **a. 視覚的調和（バランス）** | 視覚的に調和の取れた、構図の取れた | (芸術作品の内容・構図が)奇妙な |
|  | **b. 奥行** | 2次元的 | 3次元的 |
|  | **c. 複雑性** | 理解が容易 | 複雑 |
|  | **d. 色の彩度** | 柔らかい、パステル | 激しい、力強い |
|  | **e. 色のバリエーション** | 色が数ない | 色が多様 |
|  | **f. 色温度** | 暖かい色 | 冷たい色 |
|  | **g. 色世界** | 暗い | 明るい |
|  | **h.** 筆づかい | 筆づかいが細かい | 筆づかいが粗い |
|  | **i. 描画領域** | 余白が多い | 画面が満たされている |
| **ii. コンテンツ属性** | **j. 抽象性** | 具象的 | 抽象的 |
|  | **k. 想像性** | 現実的な内容/トピック | 想像的、非現実的、幻想的 |
|  | **l. 象徴性（あいまいさ）** | 明確（解釈が明確である） | 象徴的（多様な解釈が可能な） |
|  | **m. 物体表現** | 写実的 | 芸術性の高い |
|  | **n. 躍動感、アニメーション** | 動的 | 静か |
|  | **o. 感情性** | 感情的でない | 感情的に満たされた |
|  | **p. 情動価** | ネガティブ | ポジティブ |
|  | **q. 焦点** | 外観・環境に焦点を当てた | 内容に焦点を当てた |
